# Optimisation of Ultrasound-Assisted Extraction of Total Phenolics and Flavonoids Content from *Centella asiatica*

**DOI:** 10.3390/foods14020291

**Published:** 2025-01-17

**Authors:** Vimolpa Hiranpradith, Nantawan Therdthai, Aussama Soontrunnarudrungsri, Oumaporn Rungsuriyawiboon

**Affiliations:** 1 Department of Product Development, Faculty of Agro-Industry, Kasetsart University, Bangkok 10900, Thailand; vimolpa.hi@ku.th (V.H.); aussama.s@ku.th (A.S.); 2 Department of Veterinary Technology, Faculty of Veterinary Technology, Kasetsart University, Bangkok 10900, Thailand; cvtopr@ku.ac.th

**Keywords:** extraction, ultrasound, *Centella asiatica*, polyphenol, flavonoid, optimisation, model, RSM

## Abstract

*Centella asiatica* (CA), known for its health-promoting properties, is rich in bioactive compounds. This study optimised ultrasound-assisted extraction (UAE) parameters to maximise total phenolic content (TPC) and total flavonoid content (TFC) using the response surface methodology (RSM). Ethanol concentration and solvent volume significantly influenced TPC and TFC yields (*p* < 0.0001), while ultrasonic power had nonsignificant effects (*p* < 0.05). Time showed no significant effect on TPC (*p* > 0.05) but influenced TFC due to flavonoids’ sensitivity to degradation (*p* < 0.05). Variable interactions were negligible (*p* > 0.05). The relationship between responses (TPC and TFC) and independent parameters could be expressed as the quadratic models fitted with a Predicted R^2^ of 0.8263 for TPC and 0.9006 for TFC. Based on RSM, the optimal conditions—75% ethanol concentration, 87.5 W ultrasonic power, 30 min extraction time, and 20 mL solvent volume—yielded TPC and TFC values of 52.29 ± 1.65 mg/g and 43.71 ± 1.92 mg/g, closely aligning with model predictions at 95% confidence. Additionally, the optimal UAE condition provided asiaticoside of 37.56 ± 4.25 mg/g and madecassoside of 16.91 ± 1.28 mg/g. This study offers valuable insights into the factors influencing UAE efficiency, sustainability, and scalability for recovering bioactive compounds, underscoring its potential as a sustainable method for developing functional food ingredients from CA.

## 1. Introduction

*Centella asiatica* (CA), a medicinal plant belonging to the *Apiaceae* family, is widely recognised for its diverse pharmacological properties and traditional medicinal applications. Known by various names, such as Gotu kola, Indian pennywort, and Buabok, CA has been historically utilised to treat a wide range of conditions, including skin disorders, inflammation, metabolic dysfunctions, memory decline, and gastrointestinal issues [1,2,3,4]. Beyond its medicinal value, CA is extensively consumed as a functional food in countries such as Sri Lanka, Malaysia, India, and Thailand [5]. For instance, in Sri Lanka, CA is a key ingredient in traditional dishes such as Kola Kenda—a herbal gruel [6,7]—and Gotu Kola Sambola, a salad known to enhance dietary fibre intake and glycaemic control [8]. In Malaysia, it is consumed raw as part of ulam, a traditional Malay salad, while in Vietnam, Thailand, and India, it is incorporated into salads, cold rolls, juices, and tonics [9]. Herbal teas and beverages derived from CA are also popular for their antioxidant properties [10,11], with studies showing positive effects on gastrointestinal health, probiotic balance, and mental alertness [12,13]. Moreover, CA has been innovatively used to fortify chocolate [14] and noodles [15] to boost their nutritional profile. Notably, its inclusion in yoghurt formulation has been shown to improve antioxidant activity, phenolic content, flavonoid levels, and antidiabetic properties by inhibiting alpha-amylase enzyme activity in vitro [16].

The bioactivity of CA is attributed to its rich array of secondary metabolites, including triterpenes, flavonoids, and phenolic acids, which exhibit potent anti-inflammatory, antioxidant, neuroprotective, and wound-healing properties [17,18]. Among these compounds, triterpenes such as asiaticoside and madecassoside are recognised as biomarkers of CA’s therapeutic potential [19]. Furthermore, polyphenols and flavonoids—key subsets of CA’s bioactive constituents—are widely acknowledged for their ability to mitigate oxidative stress, inflammation, and age-related degenerative diseases, including cardiovascular, neurological, and metabolic disorders [20,21,22]. Despite the growing recognition of their health benefits, research on CA has predominantly focused on triterpenes, with limited attention to the synergistic roles of total phenolic content (TPC) and total flavonoid content (TFC) in its extracts [1,23].

Ethanolic extracts of CA, rich in TPC, TFC, and antioxidants, have demonstrated significant antimicrobial activity against foodborne pathogens and spoilage microorganisms, including *Bacillus cereus*, *Escherichia coli*, *Salmonella enterica*, *Staphylococcus aureus*, *Aspergillus niger*, and *Candida albicans* [24]. Additionally, phenolic-rich extracts of CA have shown potent radical-scavenging activity and a neuroprotective role against transthyretin amyloidosis, further underscoring their therapeutic relevance [25]. The demonstrated correlation between TPC and triterpenes necessitates a further exploration of their combined contributions to CA’s therapeutic potential [26,27,28,29,30].

Solvent extraction remains the most widely employed method for recovering bioactive compounds from CA. However, conventional methods, such as maceration, decoction, reflux, and Soxhlet extraction, are associated with significant drawbacks, including the use of large volumes of toxic solvents, extended processing times, and high energy demands [31,32]. These limitations have prompted a shift toward green extraction technologies that adhere to the principles of green chemistry by reducing solvent consumption, minimising energy usage, and preserving heat-sensitive compounds [33]. Among these methods, ultrasonic-assisted extraction (UAE) has emerged as a promising alternative. UAE utilises acoustic cavitation to disrupt cell walls and enhance mass transfer, facilitating the efficient recovery of bioactive compounds under mild conditions. Compared to conventional methods, UAE operates at lower temperatures and shorter durations, reducing compound degradation while achieving higher yields. Additionally, its solvent and energy reductions address environmental concerns [34].

The efficiency of UAE is influenced by several key parameters, including ethanol concentration, ultrasonic power, extraction time, and solvent volume. The optimisation of these variables is critical for maximising the yields of bioactive compounds and advancing UAE as a sustainable extraction approach [31]. While previous studies have explored UAE for extracting bioactive compounds from CA, reported outcomes and optimal conditions vary considerably [32]. For example, some studies have identified ethanol or methanol as the most effective solvents [35,36], while others emphasised different solvents, solid-to-solvent ratios, ultrasonic power levels, extraction temperatures, and extraction durations as critical factors [37,38]. These inconsistencies highlight the need for a more comprehensive understanding of how individual parameters influence UAE efficiency, particularly in the context of TPC and TFC extraction from CA.

This study aims to address these gaps by optimising UAE conditions for the efficient extraction of TPC and TFC from *C. asiatica*. Using response surface methodology (RSM), a robust statistical tool for process optimisation, our study investigates the effects of four key variables—ethanol concentration, ultrasonic power, extraction time, and solvent volume—on extraction efficiency. By elucidating the mechanisms underlying UAE performance, this research seeks to establish a reproducible framework for maximising bioactive compound recovery under green extraction.

## 2. Materials and Methods

### 2.1. Materials

#### 2.1.1. Plant Materials

The aerial parts of *Centella asiatica* spp. (CA) were obtained from a local farmer community enterprise in Sai Noi, Nonthaburi, Thailand. The plant material was authenticated, and a voucher specimen (#BK085866) was deposited at the Bangkok Herbarium, Plant Varieties Protection Department of Agriculture, Chatuchak, Thailand. The material was thoroughly washed and dried in a hot air oven at 40 °C for 24 to 48 h until a consistent weight was achieved. Subsequently, the dried material was ground into powder (60 mesh sieved) using a rotor mill (Hosokawa Micron, Model AP-S, Osaka, Japan). The resulting plant powder was stored in metallised packaging and kept in a desiccator at room temperature for future use.

#### 2.1.2. Chemicals and Reagents

Standard asiaticoside and madecassoside were acquired from Chromadex (Los Angeles, CA, USA), Standard quercetin and gallic acid, including Folin–Ciocalteu reagent were from Sigma–Aldrich Co., Ltd. (Taufkirchen, Germany). Analytical grade absolute ethanol (QRëC, Aucland, New Zealand) was secured from a local vendor. Methanol (HPLC grade) and ultrapure water (HPLC grade) were purchased from Fisher Scientific™ (Loughborough, UK). Orthophosphoric acid (HPLC grade) was purchased from Loba Chemie™ (Mumbai, India). Other chemicals, such as sodium carbonate (Na_2_CO_3_), Aluminium chloride (AlCl_3_), and Potassium acetate (CH_3_COOK), were from KemAus™ (Cherrybrook, Australia).

### 2.2. Methods

#### 2.2.1. Ultrasonic Extraction of CA

CA powder (0.5 g) was mixed with solvent in a 7.5 × 3.2 cm. cylindrical glass tube. The tube was then placed in an ultrasonic water bath (16.5 × 26.5 × 22.5 cm^3^; or 3.2 L capacity; 40 kHz; 30–120 W, Shenzhen, China). During the extraction process, the temperature was monitored every 5 min using a thermometer to study the effect of different ultrasonic power levels and time on temperature during the UAE. Following extraction, the supernatant was separated via centrifugation at 4000 rpm for 15 min, then filtered through Whatman filter paper (0.45 μm). The resulting clear liquid containing the target compounds was stored in an amber centrifuge tube and kept in a refrigerator for subsequent analysis on the same day.

#### 2.2.2. Experimental Design for Response Surface Methodology

A central composite design (CCD) was employed to explore the relationship between process variables and response outcomes, aiming to optimise the extraction process of total phenolic content and total flavonoid content from CA using response surface methodology (RSM). The independent variables included ethanol concentration (*X*_1_: 0 to 100%), ultrasonic power (*X*_2_: 0 to 100% or 0 to 120 W), extraction time (*X*_3_: 10 to 90 min), and solvent volume (*X*_4_: 5 to 25 mL per 0.5 g sample). The response variables were TPC (*Y*_1_) and TFC (*Y*_2_). The selection and range of these factors were based on a thorough review of the literature [37,38,39]. Each independent variable was evaluated at five coded levels: −α, −1, 0, +1, and +α, as shown in Table 1.

Equation (1) represents a second-order polynomial model applied to predict the response parameters

*Y_k_* = β_0_ + ∑_i=1_(β_i_*X*_i_) + ∑_i=1_(β_ii_*X*_i_^2^) +∑_i=1_∑_j=i+1_(β_ij_ *X*_i_*X*_j_), (1)

where *Y_k_* is the response parameters, *X_i_* and *X_j_* are the independent parameters, *β*_0_ is the intercept, and *β_i_*, *β_ii_*, and *β_ij_* are the regression, quadratic and interaction coefficients, respectively.

#### 2.2.3. Determination of Total Polyphenols and Flavonoids Content

TPC of CA extracts was measured using a modified Folin–Ciocalteu method [27]. A reaction mixture containing 1.5 mL of 10% (*v*/*v*) Folin–Ciocalteu reagent and 0.3 mL of the extract was incubated in the dark for 10 min at room temperature. Afterwards, 1.2 mL of 7.5% (*w*/*v*) sodium carbonate (Na_2_CO_3_) solution was added, vortexed, and incubated in the dark for another 30 min. The absorbance was recorded at 765 nm using a spectrophotometer (Shimadzu, Kyoto, Japan). A gallic acid standard curve was used to calculate the TPC, expressed as milligrams of gallic acid equivalents per gram of sample (mg GAE/g). Each sample was analysed in triplicate.

The total flavonoid content (TFC) was quantified using a modified aluminium chloride colourimetric assay [27]. Briefly, a reaction mixture of 0.75 mL methanol, 0.05 mL 10% (*w*/*v*) aluminium chloride (AlCl_3_), 0.05 mL 1 M potassium acetate, and 1.4 mL distilled water was combined with 0.25 mL of the extract. The mixture was incubated at room temperature for 30 min. The absorbance was measured at 420 nm using a spectrophotometer. A quercetin standard curve was used to determine the TFC, expressed as milligrams of quercetin equivalents per gram of sample (mg QE/g). All analyses were conducted in triplicate.

#### 2.2.4. Validation of the Model

The TPC and TFC from the CA extract were reevaluated in triplicate under the recommended optimum conditions and then compared with the predicted values to determine the validity of the model at 95% confidence (*p* < 0.05).

#### 2.2.5. Identification of Asiaticoside and Madecassoside by the High-Performance Liquid Chromatography (HPLC)

In addition to polyphenols, triterpenes such as asiaticoside (AS) and madecassoside (MS) are key bioactive compounds in CA and are widely recognised for their health-promoting properties. To quantify these triterpenes under optimal extraction conditions and compare them with extractions using 0% ethanol and 50% ethanol (75 W ultrasonic power, 50 min, 15 mL solvent volume), HPLC was conducted as described by Monton, Settharaksa [40].

The analysis focused on MS and AS, utilising a reversed-phase HPLC system (Agilent 6420, Agilent Technologies, CA, USA) equipped with an autosampler and a photodiode array detector. Before the analysis, samples were filtered through a 0.22 µm nylon filter and diluted with methanol. The prepared samples were injected into a guard column (4.0 mm × 2.0 mm) connected to a Kinetex 2.6 µm EVO C18 column (100 mm × 2.10 mm, Phenomenex, Torrance, CA, USA).

The mobile phase consisted of 0.01% aqueous phosphoric acid (solvent A) and acetonitrile (solvent B). A linear gradient elution was employed as follows: 20% solvent B for 2.5 min, increased to 37.5% over 7 min, further increased to 45% over 3 min and held for 10 min, followed by a decrease to 20% in 1 min, and maintained at 20% until 28 min before the subsequent injection. The flow rate of the mobile phase was set at 1 mL/min, and the injection volume was 20 µL. The detection wavelength was 210 nm. Quantification of MS and AS was conducted using calibration curves prepared with eight reference standard points ranging from 5 to 150 µg/mL, measured at 210 nm.

#### 2.2.6. Statistical Analysis

The experimental design and statistical analyses were conducted using Design-Expert^®^ software version 13.0. Response surface methodology (RSM) and analysis of variance (ANOVA) were applied to evaluate the regression model coefficients, assess the statistical significance of the model terms (*p* < 0.05), and determine the model fitness. These analyses aimed to optimise UAE parameters to achieve the maximum TPC and TFC from the CA extract.

Model adequacy was evaluated through regression analysis (*R*^2^) ANOVA (*p* < 0.05). The significance of the regression model coefficients was determined using *t*-tests, with nonsignificant coefficients systematically excluded based on Akaike’s Information Criterion (AICc) forward selection, resulting in a reduced and more efficient model. Relationships between the independent parameters (*X*_1_, *X*_2_, *X*_3_, and *X*_4_) and the response variables (*Y*_1_ and *Y*_2_) were visualised through response surface plots.

## 3. Results and Discussion

A central composite design within the framework of response surface methodology was utilised to optimise the key parameters for the UAE of TPC and TFC from CA. The independent parameters investigated were ethanol concentration (*X*_1_), ultrasonic power (*X*_2_), extraction time (*X*_3_), and solvent volume (*X*_4_). A total of 30 experimental conditions were conducted, with TPC and TFC values ranging from 7.39 to 53.82 mg GAE/g and 6.23 to 43.71 mg QE/g, respectively, as summarised in Table 2.

Compared to conventional extraction methods like maceration, infusion, and decoction, UAE significantly reduces the extraction time, energy consumption, and solvent usage while preserving heat-sensitive compounds [41,42,43]. From this study, the lowest TPC from aqueous-extracted CA using UAE (7.39 mg/g) were three times higher than those from water-extracted CA using cold maceration (1:20 solid-to-solvent ratio, 24 h) (2.86 mg/g) [44]. In addition, Rahman, Hossain [45] reported TPC values of 45.2 µg/mg using Soxhlet extraction with 50% ethanol at 45 °C over four cycles, which were in the same range as the results obtained here but with longer time and energy used. Moreover, a previous study by Gunathilake, Ranaweera [46] reported an optimal TPC yield of 4.71 mg/g under maceration extraction conditions (37% ethanol, 70.2 °C, 110.5 min, 20 mL solvent), which is nearly tenfold lower than the maximum TPC yield (53.82 mg/g) achieved in this study under UAE conditions (75% ethanol, 70 min, lower energy input; Table 2). Similar to our study, the maceration study also showed minimal increases in phenolic yields with longer durations and higher temperatures (e.g., 3.31 to 4.13 mg GAE/g). However, this was attributed to surface-level dissolution rather than the efficient extraction of bound phenolics, highlighting the limitations of conventional methods. In contrast, UAE generates cavitation bubbles that collapse and produce microjet turbulence, disrupting plant cell membranes. This process enhances solvent penetration and facilitates the release of bioactive compounds.

Compared to other green extraction technologies, such as microwave-assisted extraction (MAE), Sabaragamuwa and Perera [30] reported TPC and TFC values of 13.47 mg/g and 10.28 mg/g, respectively, using MAE with methanol at 600 W microwave power, 70 °C, and a 20 min extraction time. These values were notably lower than the TPC and TFC yields in this study under UAE. Although direct comparisons are complicated by differing extraction conditions in prior research, these findings affirm UAE as an efficient and sustainable method for extracting bioactive compounds, offering advantages in reduced extraction time, higher yields, and lower environmental impact [30,35,47,48].

### 3.1. Impact of Extraction Parameters on TPC and TFC

**Figure 1:**
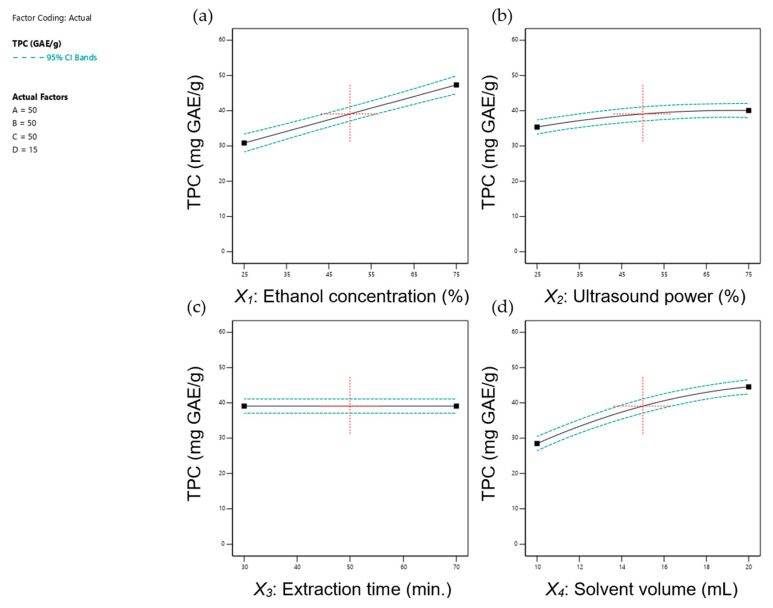
Correlation between each independent parameter including (**a**) ethanol concentration, (**b**) ultrasonic power, (**c**) extraction time, and (**d**) solvent volume on TPC under fixed conditions (50% ethanol, 50% ultrasonic power (75 W), 50 min, and 15 mL solvent volume). For uniformity, the red-dotted cross marks the central point in each graph, while unplotted factors were maintained at the model’s midpoint.

**Figure 2:**
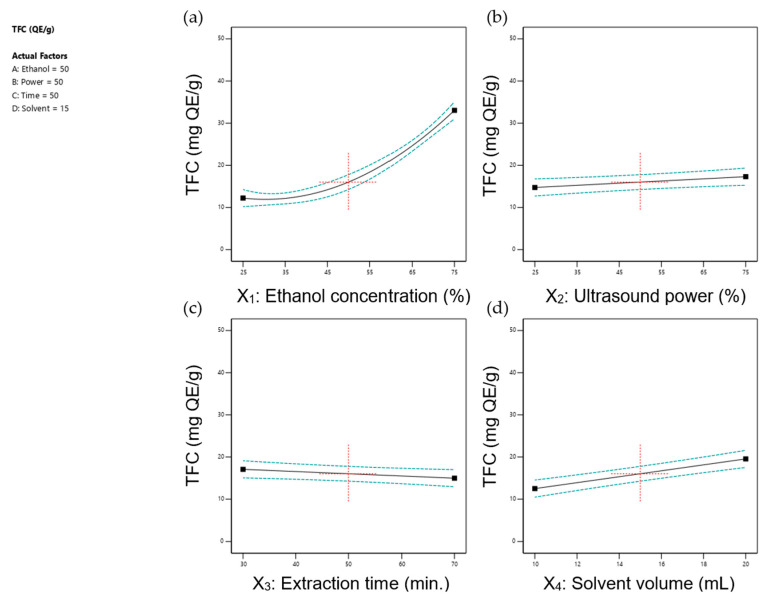
Correlation between each independent parameter including (**a**) ethanol concentration, (**b**) ultrasonic power, (**c**) extraction time, and (**d**) solvent volume on TFC under fixed conditions (50% ethanol, 50% ultrasonic power (75 W), 50 min, and 15 mL solvent). For uniformity, the red-dotted cross marks the central point in each graph, while unplotted factors were maintained at the model’s midpoint.

Figure 1 and Figure 2 depict the effects of individual extraction parameters—(a) ethanol concentration, (b) ultrasonic power, (c) extraction time, and (d) solvent volume—on the ultrasonic-assisted extraction of TPC and TFC under fixed conditions (50% ethanol, 75 W ultrasonic power, 50 min extraction time, and 15 mL solvent volume). The data indicated that most parameters positively influenced TPC and TFC yields, while prolonged extraction time had a negative impact. These findings are consistent with prior research demonstrating the complex interplay between UAE parameters and extraction yields of bioactive compounds, as detailed below.

Ethanol concentration significantly affected the extraction efficiency of TPC (*p* < 0.0001) and TFC (*p* < 0.0001). As shown in Figure 1a and Figure 2a, both TPC and TFC increased with ethanol concentration, peaking at 75%. This trend was consistent with earlier studies [46,49,50], emphasising the importance of solvent polarity in polyphenol extraction [51].

Research has established the superiority of water-ethanol mixtures over pure water or absolute ethanol for extracting polyphenols due to their synergistic interactions [52,53,54]. In these systems, water swells plant tissues, enhancing the solvent-solid interaction surface area, while ethanol facilitates polyphenol desorption and solubility. This combination lowers the dielectric constant, disrupts hydrogen bonding, and creates a more polar environment, allowing for the extraction of both high- and low-polarity polyphenolic compounds [53,55]. Additionally, water reduces ethanol’s viscosity, improving cavitation efficiency and overall UAE performance [39,56]. However, at excessively high ethanol concentrations, the extraction rate decreases due to competition between alcohol-soluble pigments and lipophilic components with ethanol-water molecules, limiting flavonoid binding and reducing extraction efficiency [57].

Flavonoid extraction, in particular, benefits from solvents with lower polarity, as reflected by the sharp increase in TFC at ethanol concentrations above 50% (Figure 2a). This observation underscores the critical role of solvent composition in optimising flavonoid recovery [55].

Ultrasonic power plays a crucial role in the UAE by generating high temperatures and pressure within collapsing cavitation bubbles. This process creates high-speed solvent jets, rupturing cell walls and enhancing extraction efficiency. The energy from ultrasonic waves is converted into kinetic energy during bubble collapse, producing heat and shear forces that promote cellular structures’ breakdown [58]. However, excessive ultrasonic power can degrade bioactive compounds due to elevated temperatures and reduced cavitation bubble formation, ultimately decreasing extraction efficiency [39,59].

**Figure 3:**
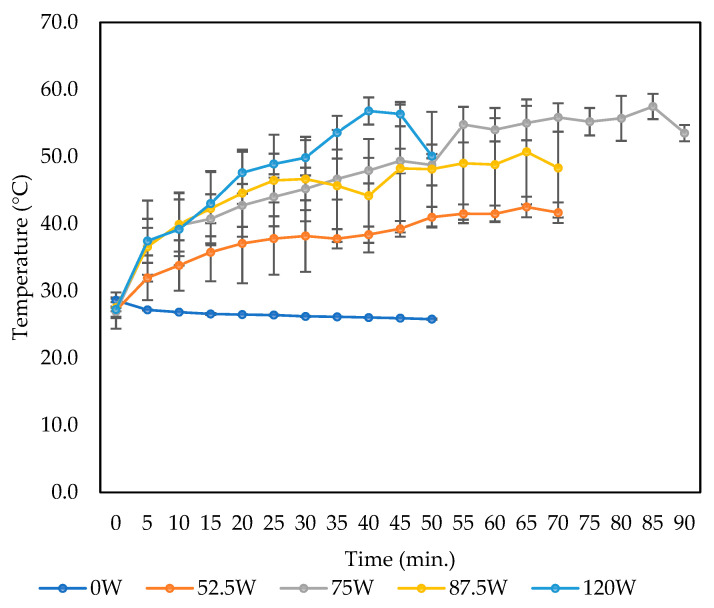
Temperature change of mixed solvent under various ultrasonic powers (0 to 120 W) during ultrasonic extraction of CA for 90 min (data are presented as mean ± SD).

In this study, temperature increases were observed with higher ultrasonic power, ranging from 25.6 °C to 55.8 °C (Figure 3). Previous research suggests that moderate temperature elevations enhance phenolic extraction by breaking matrix bonds, altering membrane structures, and reducing solvent viscosity and surface tension. However, temperatures exceeding optimal levels degrade phenolic compounds through chemical or enzymatic reactions [51,55].

TPC and TFC yields, from our study, increased slightly with rising ultrasonic power (Figure 1b and Figure 2b). The absence of a downward trend at higher power levels may be due to the temperature remaining below 60 °C, which is the threshold for optimal TPC and TFC recovery in many studies [60,61]. Additionally, the ultrasonic bath method used in this study transferred less power and energy to the sample compared to the probe method due to energy losses occurring within the medium [59].

Regarding extraction time (Figure 1c and Figure 2c), it significantly influenced TFC (*p* < 0.05) but not TPC (*p* > 0.05), aligning with the findings by Gunathilake, Ranaweera [46]. Prolonged extraction times reduced TFC, likely due to flavonoid degradation at elevated temperatures [55]. Similarly, Yang and Zhang [62] observed rapid increases in rutin and quercetin yields during the first 30 min of ultrasonic extraction, followed by diminished returns, highlighting the time-sensitivity of flavonoid recovery and the risk of degradation with extended durations. In contrast, the stability of TPC observed in this study may be attributed to the differing thermal degradation thresholds of polyphenols, which depend on their temperature sensitivity and polarity [63]. Throughout the extraction process, ultrasonic power levels (0 to 120 W) kept temperatures below 60 °C, even during the longest extraction time of 90 min (Figure 3). This temperature likely minimised the thermal degradation of polyphenols. Our findings align with those of Albuquerque, Pinela [64], who reported that while extraction time alone did not significantly affect anthocyanin, the extraction performance was optimal when high ultrasonic power was applied for shorter durations. Prolonged high-power sonication, however, led to a decrease in anthocyanin content, whereas lower ultrasonic power required extended times to achieve effective extraction. They also noted that high yields of anthocyanins could be obtained with ultrasonic power > 376 W and processing times > 13 min. However, it should be noted that high ultrasonic power could degrade the polyphenol compounds due to oxidation.

In our study, although ultrasonic power facilitates cavitation and cell wall disruption, the energy transfer under the experimental conditions (0 to 120 W) may have been insufficient to significantly accelerate the extraction process. As a result, the polyphenols likely remained stable, reflecting early-phase extraction kinetics, as proposed by Sen, Chouhan [65].

In addition, TPC (Figure 1d) and TFC (Figure 2d) increased with solvent volume, consistent with prior studies [53,59]. Adequate solvent volume facilitates hydration, enhances cavitation, promotes mass transfer, and improves compound diffusion. However, excessive solvent usage can increase costs, generate waste, and lead to impurity dissolution, ultimately reducing extract yields [55,57]. While previous research suggested a liquid-to-solid ratio of 25:1 for UAE of bioactive compounds from *C. asiatica* using methanol [30,35], the highest TPC and TFC yields in this study were achieved at a ratio of 40:1 (20 mL solvent volume). Differences in experimental variables such as solvent type, extraction conditions, sonicator type, temperature, and duration likely account for this variation.

### 3.2. Model Fitting

#### 3.2.1. Model for Prediction of TPC

Analysis of variance (ANOVA) was conducted to evaluate the significance of the second-order polynomial equation fitted to the data. Initially, the results indicated that the quadratic model was statistically significant (*p* < 0.0001) with a nonsignificant lack of fit (*p* > 0.05). However, a notable discrepancy was observed between the Adjusted R^2^ (0.7520) and Predicted R^2^ (0.1713), suggesting limitations in the model’s predictability. 

**Figure 4:**
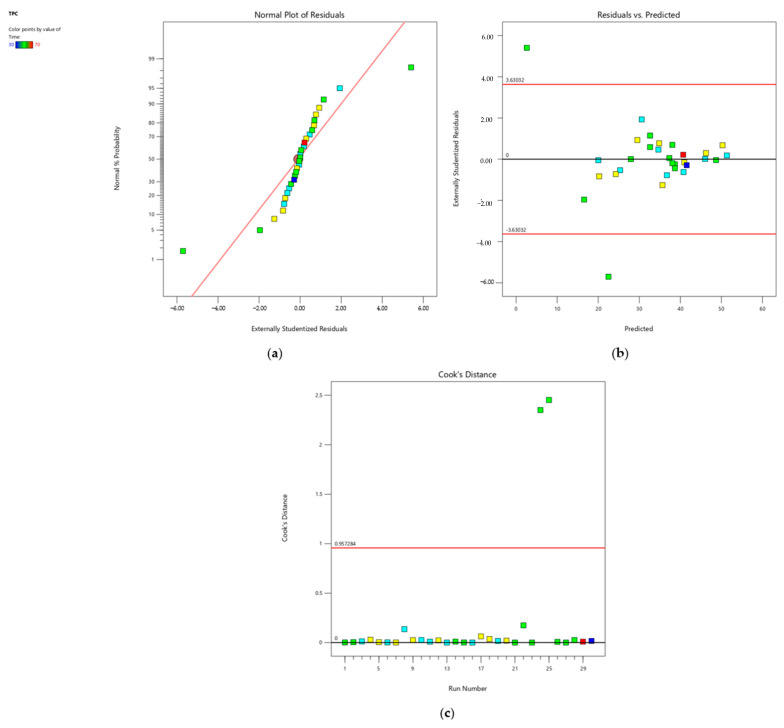
Diagnostic plots of TPC extraction from CA under UAE. (**a**) Normal plot of residuals; the red line illustrates a theoretical normal distribution line, (**b**) residuals vs. predicted plot, and (**c**) Cook’s Distance plot.

To address this issue, diagnostic plots were examined. The normal plot of residuals (Figure 4a) revealed a nonlinear pattern (S-shaped curve), indicating nonnormality in the error terms. Additionally, the residuals versus the predicted plot (Figure 4b) identified two outliers, experiment #24 and #25, which represented extreme values for ethanol concentration (*X*_1_). Cook’s Distance (Figure 4c), a measure of an observation’s influence on model estimates, exceeded 1 for these experiments, highlighting their significant impact on the predicted values.

To enhance the model’s performance, these outliers were excluded from the analysis. Following this adjustment and the removal of nonsignificant terms, the revised model was statistically significant (*p* < 0.0001) with a nonsignificant lack of fit (*p* > 0.05). The discrepancy between Adjusted R^2^ and Predicted R^2^ (0.8263) was reduced to less than 0.2, indicating an improved and reasonable model fit (Table 3).

#### 3.2.2. Model for Prediction of TFC

For TFC, ANOVA initially identified a significant linear model (*p* < 0.0001) with a nonsignificant lack of fit (*p* > 0.05). The Adjusted R^2^ was 0.7457, and the Predicted R^2^ was 0.6437. However, upon the removal of the two outliers, the quadratic model demonstrated statistical significance (*p* < 0.0001) with a nonsignificant lack of fit (*p* > 0.05). The revised model showed improved performance, with the Adjusted R^2^ increasing to 0.9404 and the Predicted R^2^ rising to 0.9006, indicating a high correlation of the predicted model (Table 3).

#### 3.2.3. Finalized Models

Table 3 presents the significant variables for the ultrasonic extraction of TPC and TFC from CA. Using the Akaike’s Information Criterion (AICc) forward selection, all observed variables were significant for both TPC and TFC extraction (*p* < 0.05), except for time in TPC extraction (*p* > 0.05). While no significant interaction effects were identified for TPC (*p* > 0.05), the interaction between ethanol concentration and solvent volume was significant for TFC (*p* < 0.05). Regarding quadratic terms, ultrasonic power and solvent effects were significant for TPC (*p* < 0.05), whereas ethanol concentration was the only significant quadratic term for TFC extraction (*p* < 0.0001).

The coefficient analysis revealed that ethanol concentration, ultrasonic power, and solvent volume positively influenced TPC yields, with ethanol concentration and solvent volume exerting the greatest effects. However, excessive levels of ultrasonic power and solvent volume led to reduced yields. For TFC, ethanol concentration and its quadratic term were the most significant factors, while solvent volume had a moderate impact, and ultrasonic power contributed minimally. This suggests that flavonoids in CA are more sensitive to solvent composition than phenolic compounds.

The reduced quadratic models for predicting TPC and TFC are presented in Equations (2) and (3), respectively.

TPC = −34.9187 + 0.329925*X*_1_ + 0.313007*X*_2_ + 4.70557*X*_4_ + (−0.00219369)(*X*_2_)^2^ + (−0.103288)(*X*_4_)^2^ (2)

TFC = −18.6488 + −0.791128*X*_1_ + 0.0511578*X*_2_ + (−0.0526294)*X*_3_ + 0.205148*X*_4_ + 0.00998143*X*_1_*X*_4_ + 0.0105767(*X*_1_)^2^ (3)

where *X*_1_ is Ethanol concentration (%),

*X*_2_ is Ultrasonic power (%),

*X*_3_ is Extraction time (minute), and

*X*_4_ is Solvent volume (mL)

### 3.3. Response Surface Plots

**Figure 5:**
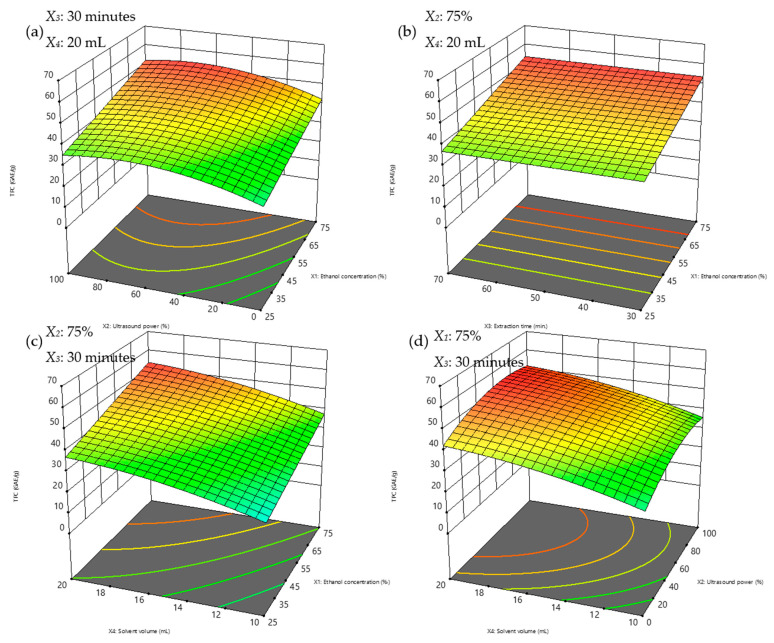
3D response surface plots illustrating the interactions affecting TPC yields under fixed conditions at (**a**) 30 min extraction time and 20 mL solvent volume; (**b**) 75% ultrasonic power (87.5 W), 20 mL solvent volume, (**c**) 30 min extraction time, 75% ultrasonic power (87.5 W); and (**d**) 30 min extraction time, 75% ethanol concentration. TPC concentrations are represented using a color gradient, ranging from blue for lower concentrations to red for higher concentrations.

**Figure 6:**
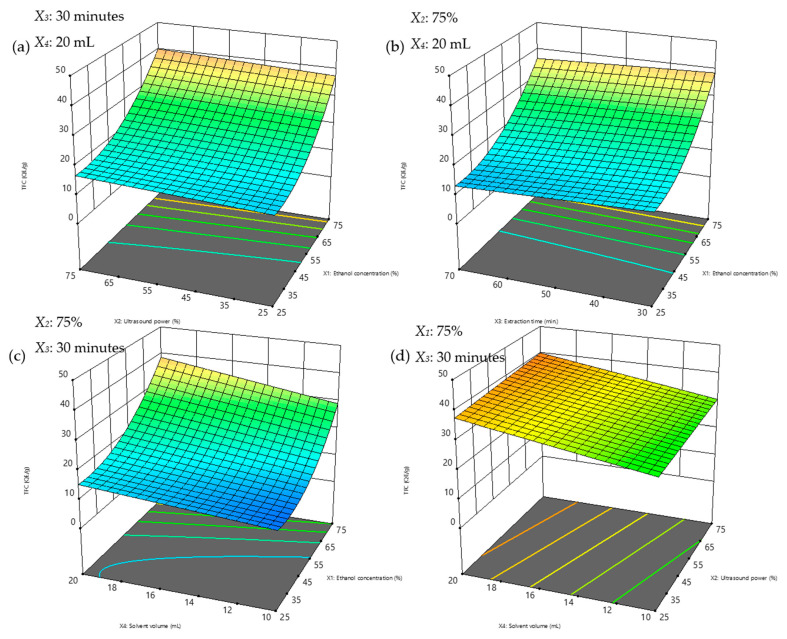
3D response surface plots illustrating the interactions affecting TFC yields under fixed conditions at (**a**) 30 min extraction time and 20 mL solvent volume; (**b**) 75% ultrasonic power (87.5 W), 20 mL solvent volume; (**c**) 30 min extraction time, 75% ultrasonic power (87.5 W); and (**d**) 30 min extraction time, 75% ethanol concentration. TFC concentrations are represented using a color gradient, ranging from blue for lower concentrations to red for higher concentrations.

Response surface analysis using three-dimensional (3D) plots visually evaluates the interactions between variables. Circular contours in the planar projection indicate insignificant interactions, while elliptical contours suggest significant interactions [66,67]. In this study, no visible elliptical or circular shapes were observed for TPC and TFC (Figure 5a–d and Figure 6a–d), confirmed by the *p*-values in Table 3, indicating no significant parameter interactions.

TPC and TFC yields peaked at the highest studied ethanol concentration (75%) without declining, suggesting the design space may have been too narrow to capture maximum yields. However, one experimental condition at 100% ethanol (Condition #25; Table 2) showed lower yields and was excluded from the model due to its negative effect on the predicted model’s precision. This isolated result underscores the need for future studies to expand the ethanol concentration range to include 100% concentration.

In line with earlier findings, ethanol concentration emerged as a critical factor in determining extraction efficiency. The synergistic effects of water and ethanol in binary solvent systems enhance the extraction of both high- and low-polarity compounds, with 50% ethanol shown to be effective for a broad range of flavonoids, while compounds such as kaempferol, quercetin, and rutin, which were found abundantly in CA [17,36,68,69,70] were recovered most from pure ethanol due to their ability to disrupt plant cell walls and enhance compound solubility [70].

Although this study did not directly analyse extraction kinetics, the results align with mechanisms proposed in prior research. Studies, such as those by Milićević, Kojić [58], have shown that the UAE extraction of polyphenols typically follows a two-stage kinetic model. The initial stage involves the rapid dissolution of surface-bound compounds, while the second stage, driven by acoustic cavitation, facilitates the release of compounds from the plant matrix through cell wall disruption. The steady TPC yields observed over time (Figure 5b) in this study suggest that the extraction process in our setup may follow a similar two-stage mechanism, with limited degradation occurring under the selected experimental conditions.

Extraction time had a minimal influence on TPC yields but showed a slight negative effect on TFC yields, potentially due to flavonoid degradation over prolonged periods. However, the controlled ultrasonic power and moderate temperatures employed in this study minimised these effects. This observation is consistent with prior research indicating that excessive sonication or prolonged extraction can lead to the thermal degradation of flavonoids [71].

Regarding solvent volume, increasing it beyond the optimal level was not advisable, as excessive solvent usage would lead to higher extraction costs, prolong the time needed for solvent removal, and diminish the advantages of reduced solvent consumption compared to conventional methods. Additionally, it would increase the environmental impact.

Although this study did not focus on solvent pH, Kruszewski and Boselli [72] highlighted its importance in influencing UAE yield and the characteristics of bioactive compounds. They noted that the optimal pH for extracting phenolic compounds typically ranges from 1 to 3. Their research also demonstrated that using an acidified binary solvent system of ethanol and water with HCl could significantly reduce the solvent-to-solid ratio from 1:20 to 1:7 without compromising anthocyanin yield. This approach suggests a promising avenue for future research to minimise solvent volume while maintaining high extraction efficiency.

### 3.4. Optimisation of UAE Parameters and Validation of the Optimal Conditions

Based on RSM, the optimal extraction parameters for maximising TPC and TFC were determined as follows: 75% ethanol concentration, 75% ultrasonic power (87.5 W), 30 min extraction time, and 20 mL solvent volume. These conditions yielded a predicted TPC of 53.76 mg/g and TFC of 40.14 mg/g. Validation experiments conducted in triplicate produced TPC and TFC values of 52.29 ± 1.65 mg/g and 43.71 ± 1.92 mg/g, respectively, closely aligning with the predicted values. These results were within the 95% confidence interval ranges (TPC: 49.28–52.29 mg/g; TFC: 36.02–44.26 mg/g), confirming the stability and reliability of the optimised extraction process. These findings validate the efficacy of the response surface methodology (RSM) model for extracting TPC and TFC from *Centella asiatica* under UAE conditions.

Under the optimal extraction conditions, both asiaticoside (AS) and madecassoside (MS) were detected at significantly higher levels of 37.56 ± 4.25 mg/g and 16.91 ± 1.28 mg/g, respectively, aligning with findings from Sabaragamuwa and Perera [30] who reported high AS (51.58 ± 0.44 mg/g) and MS (23.95 ± 0.63 mg/g) yields using UAE.

For further information, we compared the AS and MS yields with different extraction solvents, which were 0% ethanol and 50% ethanol under 75 W ultrasonic power, 50 min, and 15 mL solvent volume. The results revealed no detectable levels of AS or MS in the water-based extraction. However, in the 50% ethanol extraction, only AS was detected at 15.94 ± 1.21 mg/g. These results are consistent with previous studies [73,74,75], which reported that ethanol concentrations between 50% and 100% significantly enhance crude extract yield and pentacyclic triterpene content due to its hydrophobic nature [17].

Therefore, the findings from this study affirm the UAE’s superior performance in extracting polyphenols from CA. The scalability, sustainability, and higher efficiency of the UAE make it a promising method for bioactive compound recovery.

## 4. Conclusions

By optimising key extraction parameters, including ethanol concentration, ultrasonic power, extraction time, and solvent volume, the UAE yielded significant levels of TPC and TFC under environmentally friendly conditions. Ethanol concentration and solvent volume were identified as the primary factors influencing extraction efficiency, likely due to the “like dissolves like” principle, while ultrasonic power and time played minor roles, possibly because the extraction temperature remained controlled below 60 °C throughout the process. This study also confirmed that the UAE preserves compound integrity, offering a sustainable and scalable alternative to conventional extraction methods. These findings underscore the potential of UAE as a tool for maximising the recovery of antioxidant-rich compounds from CA, contributing to advancements in natural product extraction and applications in functional foods and nutraceuticals. Future research should explore the scalability of the optimised process, investigate alternative solvent systems, and assess the bioactivity and stability of the extracted compounds to fully understand their therapeutic potential.

## Figures and Tables

**Table 1:** Independent parameters and their coded and actual values in CCD.

Independent Parameters	Units	Symbol		Level			
			−α	−1	0	+1	*α*
Ethanol concentration (*X*_1_)	%	*X*_1_	0	25	50	75	100
Ultrasonic power (*X*_2_)	% (W)	*X*_2_	0 (0)	25 (52.5)	50 (75)	75 (87.5)	100 (120)
Extraction time (*X*_3_)	min.	*X*_3_	10	30	50	70	90
Solvent volume (*X*_4_)	mL	*X*_4_	5	10	15	20	25

**Table 2:** Recovery of the TPC and TFC from CA.

Experimental Condition	Independent Parameters *				Responses	
	*X*_1_ (%)	*X*_2_ (W)	*X*_3_ (min.)	*X*_4_ (mL)	TPC (mg GAE/g)	TFC (mg QE/g)
1	50	75	50	15	37.24	18.81
2	50	75	50	15	36.19	12.96
3	25	87.5	30	10	22.53	12.95
4	25	52.5	70	10	15.91	8.21
5	75	52.5	70	20	47.81	37.30
6	75	87.5	30	20	52.29	43.71
7	25	87.5	70	20	40.17	17.28
8	75	52.5	30	10	39.96	26.90
9	75	87.5	70	10	38.88	26.66
10	25	52.5	30	20	32.67	17.10
11	75	87.5	30	10	37.10	34.93
12	25	87.5	70	10	20.51	9.57
13	75	52.5	30	20	46.11	38.48
14	50	75	50	15	41.98	14.29
15	50	75	50	15	36.96	17.85
16	25	52.5	30	10	19.77	11.55
17	25	52.5	70	20	29.28	11.22
18	75	52.5	70	10	34.36	27.06
19	25	87.5	30	20	37.52	19.19
20	75	87.5	70	20	53.82	38.53
21	50	75	50	25	48.49	16.60
22	50	75	50	5	7.39	6.84
23	50	0	50	15	27.98	12.24
24	0	75	50	15	15.59	6.23
25	100	75	50	15	9.24	22.87
26	50	75	50	15	35.96	17.99
27	50	120	50	15	37.59	15.09
28	50	75	50	15	38.97	14.06
29	50	75	90	15	41.52	16.78
30	50	75	10	15	40.46	14.92

* Parameters are ethanol concentration (*X*_1_), ultrasonic power (*X*_2_), extraction time (*X*_3_), and solvent volume (*X*_4_).

**Table 3:** *p*-values of the independent parameters, along with the performance of the model for UAE of TPC and TFC from CA, as well as the regression coefficients of the reduced model.

Source	TPC			TFC		
	*p*-Value *		Coefficient	*p*-Value *		Coefficient
	Quadratic	Reduced Model **		Quadratic	Reduced Model **	
Model	<0.0001	<0.0001		<0.0001	<0.0001	
Intercept			39.09			16.02
*X*_1_-Ethanol (%)	<0.0001	<0.0001	8.25	<0.0001	<0.0001	10.41
*X*_2_-Power (%)	0.0021	0.0010	2.34	0.0444	0.0163	1.28
*X*_3_-Time (min.)	0.7231			0.0888	0.0429	−1.05
*X*_4_-Solvent (mL)	<0.0001	<0.0001	8.03	<0.0001	<0.0001	3.52
*X*_1_ *X*_2_	0.4386			0.7798		
*X*_1_ *X*_3_	0.6103			0.9986		
*X*_1_ *X*_4_	0.3519			0.0982	0.0493	1.25
*X*_2_ *X*_3_	0.2153			0.4585		
*X*_2_ *X*_4_	0.1277			0.7110		
*X*_3_ *X*_4_	0.3112			0.9524		
*X*_1_²	0.3869			0.0158	<0.0001	6.61
*X*_2_²	0.0506	0.0289	−1.37	0.9901		
*X*_3_²	0.5212			0.4376		
*X*_4_²	0.0026	0.0003	−2.58	0.5039		
**Lack of Fit**	0.4192	0.4146		0.2780	0.4253	
**Adjusted R²**	0.9345	0.9290		0.9195	0.9404	
**Predicted R²**	0.7818	0.8263		0.7128	0.9006	

* Terms with *p*-value > 0.05 are not significantly different at the 95% level. ** The reduced model was based on Akaike’s Information Criterion (AICc) forward selection. Abbreviations: TPC (total phenolic content), TFC (total flavonoid content).

## Data Availability

The original contributions presented in the study are included in the article, further inquiries can be directed to the corresponding author.
